# Dynamic survival analysis of gastrointestinal stromal tumors (GISTs): a 10-year follow-up based on conditional survival

**DOI:** 10.1186/s12885-021-08828-y

**Published:** 2021-11-01

**Authors:** Shao-jun Xu, Si-yu Zhang, Ling-yi Dong, Guo-sheng Lin, Yong-jian Zhou

**Affiliations:** 1 Department of Gastric Surgery, Fujian Medical University Union Hospital, No.29 Xin quan Road, Fuzhou, 350001 Fujian Province China; 2 Department of General Surgery, The First Affiliated Hospital Of Xiamen University, Xiamen, China

**Keywords:** Gastrointestinal stromal tumors, Conditional survival, Chinese patients, Prognosis, Surgery

## Abstract

**Background:**

The prognosis of patients with gastrointestinal stromal tumors (GISTs) is generally evaluated at the time of diagnosis but does not reflect the survival dynamics of patients in the future. Therefore, the purpose of this article was to evaluate the conditional survival (CS) of Chinese patients with GISTs after radical resection.

**Methods:**

This retrospective study included 451 patients who underwent radical surgery for GISTs. A Cox proportional hazard model was used to evaluate the prognostic factors of disease-free survival (DFS). The 3-year conditional DFS (CDFS_3_) of patients who survived for x years was expressed as CDFS_3=_DFS_(x + 3)_/DFS_(x)_.

**Results:**

The traditional 3-year DFS rate decreased gradually from 94.0% at 3 years to 77.3% at 7 years, while the CDFS_3_ rate increased from 94.0 to 95.2% over the survival time of the patients. In addition, classic clinicopathological prognostic factors had different effects on CDFS_3,_ with changes observed in survival time, but these effects were only slight or moderate (|d|<0.5). Although multivariate analysis showed that age, sex, mitotic index and tumor rupture were independent risk factors for DFS at baseline, all adverse prognostic factors, except for the mitotic index, lost their predictive significance at 5 years after operation. When the Modified NIH criteria were included, the risk staging was found to be an independent risk factor for recurrence or death. Time-dependent Cox regression analysis showed that the modified NIH criteria independently affected the recurrence or death of GIST patients within 2 years after operation.

**Conclusion:**

CS provides detailed dynamic survival information about Chinese patients with primary resected GISTs. The mitotic index is of great clinical significance for the monitoring and follow-up of patient populations with a high risk of tumor recurrence or death until 5 years after surgery.

**Supplementary Information:**

The online version contains supplementary material available at 10.1186/s12885-021-08828-y.

## Introduction

Gastrointestinal stromal tumors (GISTs) are the most common mesenchymal tumors of the gastrointestinal tract, accounting for 18% of all sarcomas [1–3]. A United States epidemiological study reported that the incidence of GISTs was 0.70 per 100,000 people per year between 2001 and 2015, and it has continued to increase over the last decade [4]. Complete surgical resection and postoperative adjuvant therapy with imatinib are still the only treatments for GIST patients. It has been reported that the 5-year overall survival rate of patients treated with postoperative imatinib is 83 to 92% [5, 6].

At present, the survival data of patients with GISTs are mostly evaluated from the date of diagnosis or the date of operation [7–9]. However, these studies are too dependent on static pathological factors after surgery to estimate the prognosis of patients and cannot reflect long-term survival information. Some studies have found that the risk of recurrence or death of tumor patients, including those with GIST, varies with the accumulation of survival time. More importantly, even in patients initially diagnosed with a poor prognosis, the survival rate is greatly improved after a period of survival [10–12] Therefore, the survival probability of patients cannot be accurately predicted with postoperative data, as these data can only aid in evaluating the prognosis of patients in the short term.

Conditional survival (CS) is an estimate based on the survival of patients over a period of time that can be used to obtain accurate information about their future survival time. It has been recognized as a more meaningful way to measure the probability of long-term cancer patient survival [13–15]. To the best of our knowledge, while CS has been studied with several types of malignant tumors, such as gastric cancer, ovarian cancer, pancreatic cancer and liver cancer [16–19], limited data are available on CS in Chinese patients with resected primary GISTs [10].

Although the CDFS of different races is higher than the actual DFS, the CDFS of GIST patients of different races is different across the same survival time points. However, we cannot provide valuable and accurate prognostic data for Chinese GIST patients according to the survival situation of CDFS in western countries. Moreover, to our knowledge, this study is the first to analyze the CDFS of Chinese GIST patients using data from a large sample size of patients at our center.

Therefore, the aim of our present study was to evaluate dynamic survival based on CS in patients with GISTs using a large database from China. Furthermore, we explored the effects of various clinicopathologic factors on DFS and CS in GIST patients undergoing therapeutic tumor resection.

## Methods

### Patients

This study retrospectively analyzed a prospective database containing 628 patients with GISTs from February 2002 to January 2016 at Fujian Medical University Union Hospital in China. The inclusion criteria were as follows: (1) complete clinical and pathological data; (2) pathological diagnosis of GIST; (3) endoscopic or surgical resection (R0 resection); (4) no other malignant tumors; and (5) no distant metastasis or invasion of adjacent organs (pancreas, spleen, liver, colon, etc.). In this study, 35 cases of recurrent GISTs, 79 cases of distal metastasis and 63 cases of other malignant tumors were excluded. So this retrospective study included 451 patients who underwent radical surgery for GISTs. The exclusion criteria of this study are listed in Fig. S1. The study was approved by the ethical committee of Fujian Medical University Union Hospital and all methods were performed in accordance with the relevant guidelines and regulations.

### Statistical analysis

Disease-free survival (DFS) estimates were assessed by the Kaplan-Meier method for the retrospective study. Univariable analyses were performed to assess differences in DFS between categorical groups. Cox proportional hazards models were used to assess the association of clinicopathologic information with DFS. The 3-year conditional survival (CDFS_3_) estimate is defined as the probability that a patient will survive for another 3 years after he has survived x years, indicated as CDFS_3_ = DFS(_x + 3_)/DFS(_x_) [15]. Linear regression was used to evaluate the dynamic changes in CDFS_3_, and the differences in CS between two groups were assessed by evaluating the standardized differences (d) [20]. d < |0.1| indicates very small differences between groups, |0.1| ≤ d<|0.3| indicates small differences, |0.3| ≤ d<|0.5| indicates moderate differences, and |0.5| ≤ d indicates considerable differences [11].

The d value represents the influence of various factors on prognosis, but it cannot be used to evaluate the relative independence of several predictive factors. Therefore, we used a second multivariate analysis (time-dependent multivariate analysis) to evaluate independent predictors of DFS in patients who survived a certain number of years after surgery (1, 2, 3, 4, and 5 years after operation) [21].

All data were analyzed by SPSS version 25.0 and R version 3.3.3. All tests were 2-sided, and *p* < 0.05 was recognized as significantly different.

## Results

### Demographic and clinicopathologic variables

A total of 451 patients with GISTs were included. The median age of the study subjects was 59 years (interquartile range, 20–88 years), and 46.8% of the patients were female (Table S1). The majority of patients had gastric GISTs (60.5%), while others had tumors in the small bowel (26.6%), duodenum (7.1%), and rectum (2.4%). Most patients (56.3%) had large tumors (≥ 5.0 cm), while 43.7% of patients had small tumors (< 5.0 cm). The mitotic index was less than 5.0 (per 50 HPFs) in 75.4% of patients. A small subset of patients (5.1%) had tumor rupture. Less than 4% of the patients received neoadjuvant therapy before surgery, while more than half (69.2%) of the patients received tyrosine kinase inhibitor (TKI) adjuvant therapy after resection.

### Comparison of actuarial DFS and CS

**Fig. 1:**
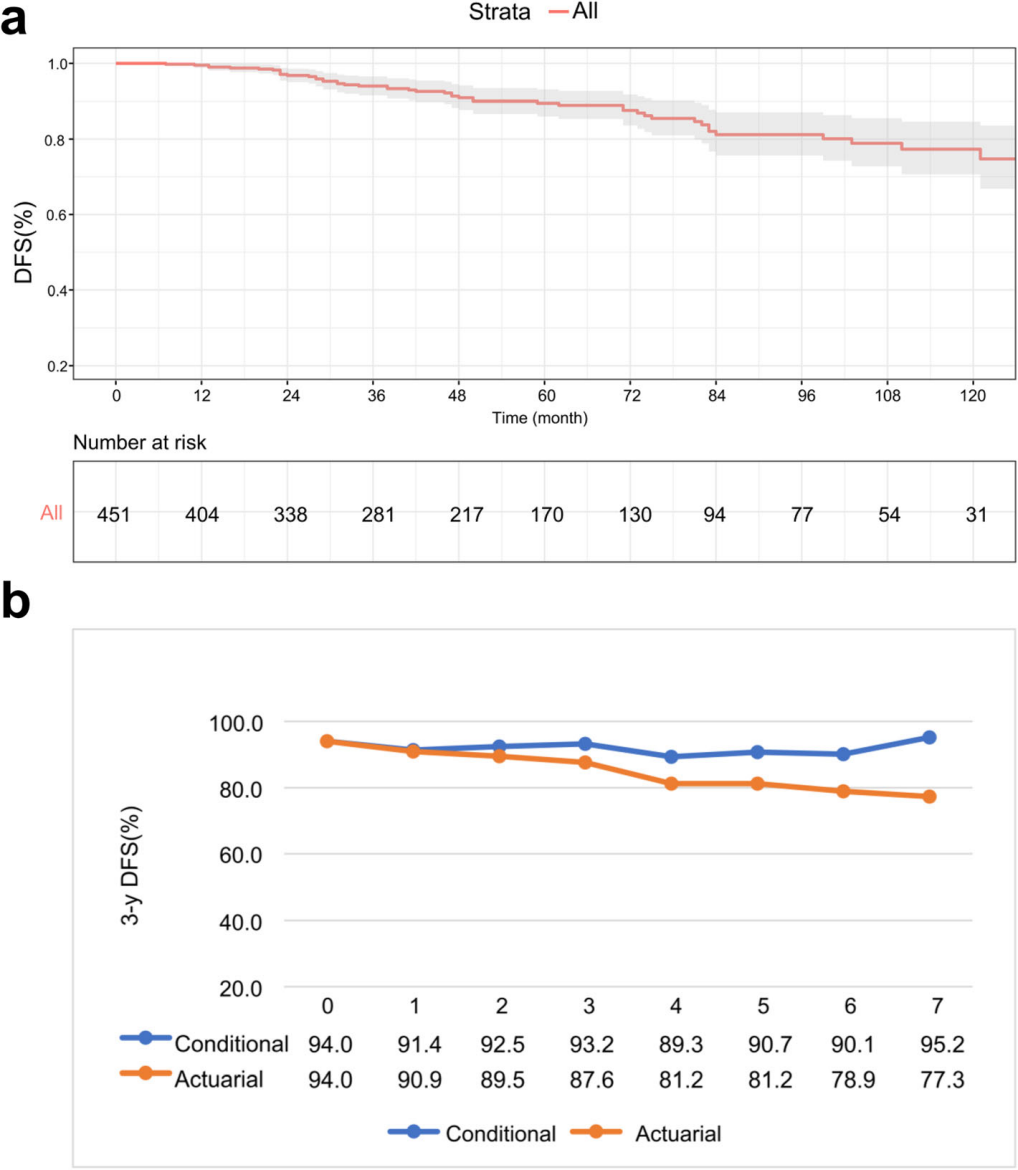
**a** Kaplan-Meier survival curve and **b** the comparison of 3-year DFS and CDFS3 over time in the whole study population

The median follow-up was 50.0 months, and within this period, 48 patients (10.6%) relapsed and died. The 3-year DFS of the study population was 94%, and the 10-year DFS was 77.3%. (Fig. 1a). Although the 3-year actuarial DFS decreased from 94.0 to 77.3% during this period, CDFS_3_ was in a stable state and tended to increase over time. For example, for patients who survived for 7 years after surgery, the CDFS_3_ of survival to 10 years was 95.2%, while the actuarial 10-year DFS was 77.3%. (Fig. 1b). Table 1 presents CS estimates of the entire cohort based on how long the patients survived after surgical resection. If the patients survived for 1 year, 2 years, 3 years, 4 years, and 5 years after surgery, then their probabilities of remaining alive at 10 years were 77.7, 79.6, 82.2, 85.0 and 86.4%, respectively.

**Table 1:** patients who reach a certain survival point given that they have already remained disease free a certain amount of time after resection of GISTs

Total survival time	If the patient has remained survived to									
	1 year	2 years	3 years	4 years	5 years	6 years	7 years	8 years	9 years	10 years
1 year	100.0									
2 years	97.6	100.0								
3 years	94.5	96.8	100.0							
4 years	91.4	93.6	96.7	100.0						
5 years	89.9	92.2	95.2	98.5	100.0					
6 years	88.0	90.2	93.2	96.4	97.9	100.0				
7 years	81.6	83.6	86.4	89.3	90.7	92.7	100.0			
8 years	81.0	83.6	86.4	89.3	90.7	92.7	100.0	100.0		
9 years	79.3	81.3	83.9	86.8	88.2	90.1	97.2	97.2	100.0	
10 years	77.7	79.6	82.2	85.0	86.4	88.2	95.2	95.2	98.0	100.0

**Fig. 2:**
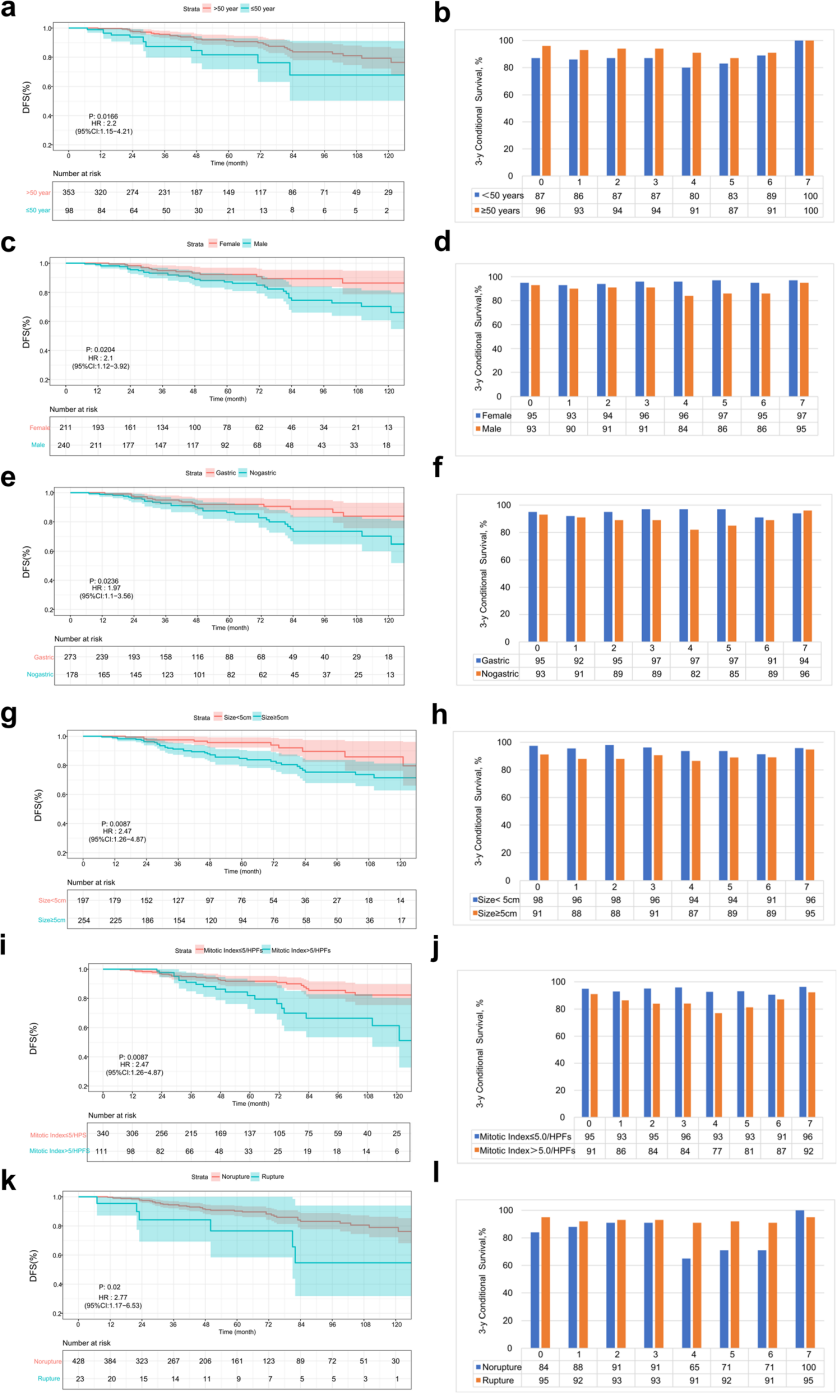
The DFS and CDFS_3_ of the patients were stratified and compared according to their sex (**a b**), age (**c d**), tumor location (**e, f**), tumor size (**g, h**), mitotic index (**i, j**), and tumor rupture status (**k, l**)

The calculated CDFS_3_ estimates were stratified by relevant demographic and tumor variables at different time points during follow-up for primary GISTs. These variables are summarized in Table 2. The effects of the different prognostic factors on DFS and CDFS_3_ over time are shown in Fig. 2. Interestingly, regarding the influence of demographic characteristics on the prognosis of GISTs, it was noted that age was relatively strongly related to poor tumor outcome at surgery (|d | = 0.34). However, its influence gradually diminished 1 year or more after operation (0.1<|d | < 0.3). In contrast, sex was a stable and limited prognostic factor during follow-up.

**Table 2:** Three-year conditional disease-free survival rates of patients in relationship to demographic and clinicopathologic characteristics

Variables	Conditional 3-yr disease-free survival, %					
	0 yr	1 yr	2 yr	3 yr	5 yr	7 yr
All patients	94.0	91.4	92.2	93.2	90.7	95.2
Age						
<50	87.3	85.5	87.0	87.3	83.0	100.0
≥ 50	95.6	93.2	93.5	94.0	87.4	100.0
**|**d|	0.34	0.28	0.24	0.26	0.13	/
Sex						
Female	94.9	93.2	93.8	95.7	96.9	96.6
Male	93.1	89.7	91.2	91.2	85.5	94.5
|d|	0.08	0.12	0.10	0.18	0.40	0.10
Tumor size, cm						
< 5.0	97.5	95.6	98.1	96.3	93.7	95.8
≥ 5.0	91.2	88.0	88.0	90.7	89.0	94.8
|d|	0.26	0.30	0.38	0.22	0.16	0.05
Tumor location						
Gastric	94.9	92.0	94.6	96.9	96.5	94.3
Nongastric	92.7	90.5	88.5	89.2	85.1	95.5
|d|	0.09	0.05	0.22	0.32	0.42	0.05
Mitotic Index, per 50 HPFs						
≤ 5.0	94.9	92.9	95.1	95.9	93.1	96.3
>5.0	91.0	86.3	83.9	84.0	81.2	92.3
|d|	0.16	0.23	0.42	0.46	0.40	0.20
Tumor rupture						
Yes	84.2	88.2	91.0	91.0	71.4	100.0
No	94.5	91.5	92.5	93.3	92.1	95.1
|d|	0.43	0.12	0.06	0.09	0.72	0.23

In our study population, tumor size and tumor location still influenced CS. The effect of tumor size on the CDFS_3_ of the patients reached the maximum at 2 years after operation (|d | = 0.38), while the tumor location effect reached the maximum value when the patients survived for 5 years (| d | = 0.42); however, in patients who survived longer, there was a slight or very small correlation between tumor size and tumor location and CDFS_3_. Throughout the study period, a mitotic index > 5.0 (per 50 HPFs) continued to affect CDFS_3_ to a moderate or strong degree.

### Prognostic factors associated with DFS over time

In the subgroup analysis, it was found that age (< 50 years), male sex, large tumor size, high mitotic index, non-gastric location and tumor rupture were associated with poor prognosis (all *p* < 0.05) (Table 3). To further clarify the independent risk factors that dynamically affect DFS, we used time-dependent multivariate analysis (Table 4). At the time of surgery, age, sex, mitotic index, and tumor rupture were independent prognostic factors for curative surgical resection of GISTs (*p* < 0.05). After 1 year of surgery, tumor rupture was not an independent risk factor for DFS, but age, sex and mitotic index were still effective predictors of DFS. In patients who had already survived for 3 years, sex and mitotic index remained independent prognostic factors (*p* < 0.05). Of note, when the patients survived 5 years after surgery, only the mitotic index was related to poor prognosis. Notably, the mitotic index lost prognostic significance after 6 years of survival.

**Table 3:** Actuarial disease-free survival rates of patients in relationship to demographic and clinicopathologic characteristics

Variables	Patient disease-free survival				
	3 years, %	5 years, %	8 years, %	10 years, %	*p*
All patients	94.0	89.5	81.2	77.3	
Age					0.014
<50	87.3	81.6	67.7	67.7	
≥ 50	95.6	91.3	83.6	79.3	
Sex					0.018
Female	94.9	92.2	89.3	86.3	
Male	93.1	87.1	74.5	70.3	
Tumor size, cm					
< 5.0	97.5	95.6	89.6	85.8	0.007
≥ 5.0	91.2	84.7	75.4	71.5	
Tumor location					0.021
Gastric	94.9	92.0	88.8	83.8	
Nongastric	92.7	86.4	73.5	70.2	
Mitotic Index, per 50 HPFs					0.003
≤ 5.0	94.9	91.8	85.5	82.3	
>5.0	91.0	81.9	66.5	61.4	
Tumor rupture					0.015
No	94.5	90.2	83.1	79.0	
Yes	84.2	76.6	54.7	54.7

**Table 4:** Time-dependent multivariate analysis of the prognostic factors for patients with GISTs

	Hazard ratio	*p*
At Surgery		
Age		0.013
≥ 50	1·00 (reference)	
<50	2.31 (1.20,4.44)	
Sex		0.031
Female	1·00 (reference)	
Male	2.00 (1.07,3.76)	
Tumor size, cm		0.152
< 5.0	1·00 (reference)	
≥ 5.0	1.69 (0.83,3.46)	
Tumor location		0.259
Gastric	1·00 (reference)	
Nongastric	1.42 (0.77,2.62)	
Mitotic Index, per 50 HPFs		0.014
≤ 5.0	1·00 (reference)	
>5.0	2.10 (1.17,3.78)	
Tumor rupture		0.046
No	1·00 (reference)	
Yes	2.41 (1.02,5.71)	
1 yr		
Age		0.025
≥ 50	1·00 (reference)	
<50	2.17 (1.10,4.26)	
Sex		0.042
Female	1·00 (reference)	
Male	1.93 (1.02,3.64)	
Mitotic Index, per 50 HPFs		0.005
≤ 5.0	1·00 (reference)	
>5.0	2.34 (1.29,4.25)	
3 yr		
Sex		0.042
Female	1·00 (reference)	
Male	1.93 (1.02,3.64)	
Mitotic Index, per 50 HPFs		0.005
≤ 5.0	1·00 (reference)	
>5.0	2.34 (1.29,4.25)	
5 yr		
Mitotic Index, per 50 HPFs		0.023
≤ 5.0	1·00 (reference)	
>5.0	3.25 (1.17,8.97)	
6 yr		
	None	

When the modified NIH staging criteria were included in the time-dependent multivariate regression analysis, only the modified NIH staging criteria and age were independent prognostic factors for baseline DFS. The modified NIH staging criteria had a sustainable effect on DFS for 2 years after operation (*p*<0.05). In general, there was no significant difference between the patients who did not experience recurrence or died and those who survived for more than 2 years across all four staging groups (*p* > 0.05)(Table S2).

## Discussion

Traditionally, the prognosis of GIST patients is estimated by survival rates for certain time points depending on a number of factors determined at the time of diagnosis [22, 23]. The modified National Institutes of Health (NIH) criteria [24] and the American Joint Committee on Cancer (AJCC) tumor-node-metastasis (TNM) staging system are the most widely used recurrence risk assessment models. However, some investigators have established nomogram-based models to individually predict long-term survival in tumor patients [7, 25]. However, these estimates are static, and oncologists and patients cannot use this simple information to obtain more accurate distant prognostic assessments.

CS is an important way to evaluate long-term survival, which takes into account not only the unique pathological characteristics of the tumor but also the survival time of the individual after tumor resection [12, 26]. Thus, CS could provide more accurate and dynamic survival information after a certain point of survival. A previous large multicenter data study in the United States assessed the CS of GISTs and showed that the CDFS of GISTs gradually improved with the extension of survival time and provided valuable survival information [27]. The results showed that the CDFS of GIST gradually improved with survival time and provided valuable survival information. However, the proportion of GIST patients who received TKI adjuvant therapy after tumor resection was only 23%. In this study, the proportion of GIST patients receiving adjuvant therapy after radical surgery was as high as 69.2%. Thus, our data can better reflect the dynamic survival of GIST patients in the era of imatinib treatment. However, these results are based on data from Western populations and are therefore lacking in terms of reference for Chinese clinicians. Therefore, exploring changes in survival among Chinese patients with GISTs in the period after surgery is highly important.

In our study, we found that the estimated CDFS exceeded the traditional DFS estimate, and this performance was more pronounced among patients with GISTs as cumulative survival time increased. For example, the difference between actuarial 3-year DFS and CDFS_3_ at 3 and 4 years after operation was approximately 6–8%; however, at 6 and 7 years, the difference was even more significant (Δ ≈ 11–18%). Consistent with previous studies [27], our study showed that the CDFS among patients with poor prognosis at surgery improved the most. This situation shows that the traditional DFS estimation can reflect only the short-term prognosis of patients after operation, while CDFS is more meaningful for long-term survival assessment. Zamboni et al. [28] described that this phenomenon quantifies the concept of the “natural selection effect”. This concept holds that newly diagnosed cancer patients are a group of people with different risks of recurrence or death. Over time, high-risk patients die in the first few years, while low-risk patients become “healthier”.

Several studies have demonstrated that tumor-related CS improves over time, especially for those patients with a poor prognosis at the time of surgery [2, 16, 19, 26]. However, in the current study, it was found that CDFS did not increase over time and that the survival rate at most time points showed a moderate decline. The lack of time-dependent changes in the prognostic significance of these factors is reasonable because patients have different tumor characteristics and degrees of tumor burden, which can have persistent adverse effects on survival that we cannot predict. There is no optimal routine follow-up strategy for GIST patients undergoing radical surgery. In some institutions, high-risk GIST patients undergo routine abdominal CT follow-up every 3–6 months during adjuvant therapy for a period of 3 years [29]. In this study, it was found that the CDFS of high-risk GIST patients increased or decreased moderately with survival time and remained in a relatively stable state for a long time, showing a good prognosis. The potential radiation exposure from conventional CT during follow-up creates a risk for secondary malignant tumor. Therefore, the interval of CT follow-up should be extended appropriately.

Previous studies have suggested that sex, age, tumor size, mitotic index, tumor location, and tumor rupture are associated with poor long-term survival of patients GISTs [8, 9, 30, 31]. Similarly, several of these variables are adverse risk factors for the survival of GIST patients. We also found that patients with large tumor size, high mitotic index and no non-gastric location had unfavorable prognosis changes over time. For example, in the analysis of GIST patients who had survived for 2 years, the tumor size significantly affected the CDFS_3_ (|d | = 0.38); at the point of subsequent survival, the tumor size was only slightly or very slightly correlated with CDFS_3_. Nonetheless, the d value can only be used to explain the effect of prognosis; thus, it cannot be used to evaluate the independence of adjusted multiple factors. Therefore, we used time-related multivariate analysis to further evaluate the independent factors affecting the prognosis of DFS.

Age, sex, mitotic index, and tumor rupture were independent risk factors for DFS of patients with GISTs at surgery, as shown in Table 4 (all *p* < 0.05). Nevertheless, only the mitotic index continually affected DFS (*p* < 0.05) when the patients survived for 5 years, which has not been reported in previous studies. In addition, this study is the first to analyze the independent risk factors that affect the dynamics of traditional DFS. The results indicated that the mitotic index can be used as a stable marker to predict recurrence or death within 5 years after operation. Therefore, we believe that GIST patients with a high mitotic index should be followed up and observed carefully for a long time. However, the known adverse prognostic factors no longer affect the survival of patients at 6 years after surgery. This indicates that the risk factors strongly influence DFS initially, but their impact on prognosis may be weakened or even lost over time. Time-dependent multivariate analysis showed that only stage continued to affect DFS at 2 years after operation (*p* < 0.05). To some extent, this reflects that the effect of the modified NIH staging criteria on survival is relatively stable, and it is worth noting that the modified NIH staging criteria also loses the ability to affect DFS after 2 years of survival.

There are some limitations to our study that should be noted. As with all single-institution retrospective studies, the study had small sample size, and the results cannot be extrapolated to the general population. However, we provided information about the dynamic survival of patients with Chinese GIST to support further studies. Information on gene mutations and their potential influence on DFS and CDFS estimates was not studied. We look forward to the publication of more rigorous prospective studies that assess whether gene mutations influence the CDFS of patients.

## Conclusions

In summary, the estimated long-term survival of Chinese patients with GISTs changes based on the time of survival. The mitotic index can be used as a predictor of GIST stability until 5 years after surgery. The modified NIH staging criteria loses the ability to reflect DFS after 2 years of survival. CS assessment can determine the dynamic DFS of patients with GISTs after radical resection, so it is of great significance for patients who undergo surveillance for recurrence.

## Supplementary Information

**Additional file 1.**

## Data Availability

The datasets used or analyzed during the current study are available from the corresponding author on reasonable request.

## Abbreviations

GISTs: gastrointestinal stromal tumors; CS: conditional survival
DFS: Disease free survival
CDFS_3_: 3-year conditional DFS
OS: Overall survival
COS: Conditional OS

## References

[1] Ducimetière F, Lurkin A, Ranchère-Vince D, Decouvelaere A-V, Péoc'h M, Istier L, Chalabreysse P, Muller C, Alberti L, Bringuier P-P (2011). Incidence of sarcoma histotypes and molecular subtypes in a prospective epidemiological study with central pathology review and molecular testing. PLoS One.

[2] Corless CL, Barnett CM, Heinrich MC (2011). Gastrointestinal stromal tumours: origin and molecular oncology. Nat Rev Cancer.

[3] Nilsson B, Bümming P, Meis-Kindblom JM, Odén A, Dortok A, Gustavsson B, Sablinska K, Kindblom LG (2005). Gastrointestinal stromal tumors: the incidence, prevalence, clinical course, and prognostication in the preimatinib mesylate era--a population-based study in western Sweden. Cancer.

[4] Patel N, Benipa B (2019). Incidence of Gastrointestinal Stromal Tumors in the United States from 2001–2015: A United States Cancer Statistics Analysis of 50 States. Cureus.

[5] DeMatteo RP, Ballman KV, Antonescu CR, Corless C, Kolesnikova V, von Mehren M, McCarter MD, Norton J, Maki RG, Pisters PWT (2013). Long-term results of adjuvant imatinib mesylate in localized, high-risk, primary gastrointestinal stromal tumor: ACOSOG Z9000 (Alliance) intergroup phase 2 trial. Ann Surg.

[6] Joensuu H, Eriksson M, Hall KS, Reichardt A, Hartmann JT, Pink D, Ramadori G, Hohenberger P, Al-Batran S-E, Schlemmer M (2016). Adjuvant Imatinib for High-Risk GI Stromal Tumor: Analysis of a Randomized Trial. J Clin Oncol.

[7] Gold JS, Gönen M, Gutiérrez A, Broto JM, García-del-Muro X, Smyrk TC, Maki RG, Singer S, Brennan MF, Antonescu CR, Donohue JH, DeMatteo RP (2009). Development and validation of a prognostic nomogram for recurrence-free survival after complete surgical resection of localised primary gastrointestinal stromal tumour: a retrospective analysis. Lancet Oncol.

[8] Gronchi A, Bonvalot S, Poveda Velasco A, Kotasek D, Rutkowski P, Hohenberger P, Fumagalli E, Judson IR, Italiano A, Gelderblom HJ, van Coevorden F, Penel N, Kopp HG, Duffaud F, Goldstein D, Broto JM, Wardelmann E, Marréaud S, Smithers M, le Cesne A, Zaffaroni F, Litière S, Blay JY, Casali PG (2020). Quality of Surgery and Outcome in Localized Gastrointestinal Stromal Tumors Treated Within an International Intergroup Randomized Clinical Trial of Adjuvant Imatinib. JAMA Surg.

[9] Joensuu H, Vehtari A, Riihimäki J, Nishida T, Steigen SE, Brabec P, Plank L, Nilsson B, Cirilli C, Braconi C (2012). Risk of recurrence of gastrointestinal stromal tumour after surgery: an analysis of pooled population-based cohorts. Lancet Oncol.

[10] Bischof DA, Kim Y, Dodson R, Jimenez MC, Behman R, Cocieru A, Fisher SB, Groeschl RT, Squires MH, Maithel SK (2015). Conditional disease-free survival after surgical resection of gastrointestinal stromal tumors: a multi-institutional analysis of 502 patients. JAMA Surg.

[11] Cucchetti A, Piscaglia F, Cescon M, Ercolani G, Terzi E, Bolondi L, Zanello M, Pinna AD (2012). Conditional survival after hepatic resection for hepatocellular carcinoma in cirrhotic patients. Clin Cancer Res.

[12] Harshman LC, Xie W, Bjarnason GA, Knox JJ, MacKenzie M, Wood L, Srinivas S, Vaishampayan UN, Tan M-H, Rha S-Y (2012). Conditional survival of patients with metastatic renal-cell carcinoma treated with VEGF-targeted therapy: a population-based study. Lancet Oncol.

[13] Henson DE, Ries LA, Carriaga MT (1995). Conditional survival of 56,268 patients with breast cancer. Cancer.

[14] Merrill RM, Henson DE, Ries LA (1998). Conditional survival estimates in 34,963 patients with invasive carcinoma of the colon. Dis Colon Rectum.

[15] Skuladottir H, Olsen JH (2003). Conditional survival of patients with the four major histologic subgroups of lung cancer in Denmark. J Clin Oncol.

[16] Wang P, Sun Z, Wang W, Deng J, Wang Z, Liang H, Zhou Z, Xu H (2018). Conditional survival of patients with gastric cancer who undergo curative resection: A multi-institutional analysis in China. Cancer.

[17] Kurta ML, Edwards RP, Moysich KB, McDonough K, Bertolet M, Weissfeld JL, Catov JM, Modugno F, Bunker CH, Ness RB, Diergaarde B (2014). Prognosis and conditional disease-free survival among patients with ovarian cancer. J Clin Oncol.

[18] Mayo SC, Nathan H, Cameron JL, Olino K, Edil BH, Herman JM, Hirose K, Schulick RD, Choti MA, Wolfgang CL (2012). Conditional survival in patients with pancreatic ductal adenocarcinoma resected with curative intent. Cancer.

[19] Spolverato G, Kim Y, Ejaz A, Alexandrescu S, Marques H, Aldrighetti L, Gamblin TC, Pulitano C, Bauer TW, Shen F, Sandroussi C, Poultsides G, Maithel SK, Pawlik TM (2015). Conditional probability of long-term survival after liver resection for intrahepatic Cholangiocarcinoma: a multi-institutional analysis of 535 patients. JAMA Surg.

[20] Burnand B, Kernan WN, Feinstein AR (1990). Indexes and boundaries for "quantitative significance" in statistical decisions. J Clin Epidemiol.

[21] Margonis G, Buettner S, Andreatos N, Wagner D, Sasaki K, Barbon C, Beer A, Kamphues C, Løes I, He J (2019). Prognostic factors change over time after hepatectomy for colorectal liver metastases: a multi-institutional, international analysis of 1099 patients. Ann Surg.

[22] Dematteo RP, Gold JS, Saran L, Gönen M, Liau KH, Maki RG, Singer S, Besmer P, Brennan MF, Antonescu CR (2008). Tumor mitotic rate, size, and location independently predict recurrence after resection of primary gastrointestinal stromal tumor (GIST). Cancer.

[23] Fujimoto Y, Nakanishi Y, Yoshimura K, Shimoda T (2003). Clinicopathologic study of primary malignant gastrointestinal stromal tumor of the stomach, with special reference to prognostic factors: analysis of results in 140 surgically resected patients. Gastric Cancer.

[24] Joensuu H (2008). Risk stratification of patients diagnosed with gastrointestinal stromal tumor. Hum Pathol.

[25] Lee CK, Goldstein D, Gibbs E, Joensuu H, Zalcberg J, Verweij J, Casali PG, Maki RG, Cioffi A, McArthur G (2015). Development and validation of prognostic nomograms for metastatic gastrointestinal stromal tumour treated with imatinib. Eur J Cancer.

[26] Kim Y, Margonis GA, Prescott JD, Tran TB, Postlewait LM, Maithel SK, Wang TS, Glenn JA, Hatzaras I, Shenoy R (2017). Curative Surgical Resection of Adrenocortical Carcinoma: Determining Long-term Outcome Based on Conditional Disease-free Probability. Ann Surg.

[27] Bischof DA, Kim Y, Dodson R, Jimenez MC, Behman R, Cocieru A, Fisher SB, Groeschl RT, Squires MH, Maithel SK (2015). Conditional disease-free survival after surgical resection of gastrointestinal stromal tumors. JAMA Surgery.

[28] Zamboni BA, Yothers G, Choi M, Fuller CD, Dignam JJ, Raich PC, Thomas CR, O'Connell MJ, Wolmark N, Wang SJ (2010). Conditional survival and the choice of conditioning set for patients with colon cancer: an analysis of NSABP trials C-03 through C-07. J Clin Oncol.

[29] D'Ambrosio L, Palesandro E, Boccone P, Tolomeo F, Miano S, Galizia D, Manca A, Chiara G, Bertotto I, Russo F (2017). Impact of a risk-based follow-up in patients affected by gastrointestinal stromal tumour. Eur J Cancer.

[30] Corless CL, Ballman KV, Antonescu CR, Kolesnikova V, Maki RG, Pisters PWT, Blackstein ME, Blanke CD, Demetri GD, Heinrich MC (2014). Pathologic and molecular features correlate with long-term outcome after adjuvant therapy of resected primary GI stromal tumor: the ACOSOG Z9001 trial. J Clin Oncol.

[31] Holmebakk T, Bjerkehagen B, Hompland I, Stoldt S, Boye K (2019). Relationship between R1 resection, tumour rupture and recurrence in resected gastrointestinal stromal tumour. Br J Surg.

