# Supplementary material for: Dynamic survival analysis of gastrointestinal stromal tumors (GISTs): a 10-year follow-up based on conditional survival
Source: BMC Cancer. 2021 Nov 1;21:1170. doi: 10.1186/s12885-021-08828-y (PMC8559392; doi:10.1186/s12885-021-08828-y)
Supplement: Supplementary file 1 — Additional file 1. [file 12885_2021_8828_MOESM1_ESM.docx]

Table S1. Demographic and clinicopathological of the study population


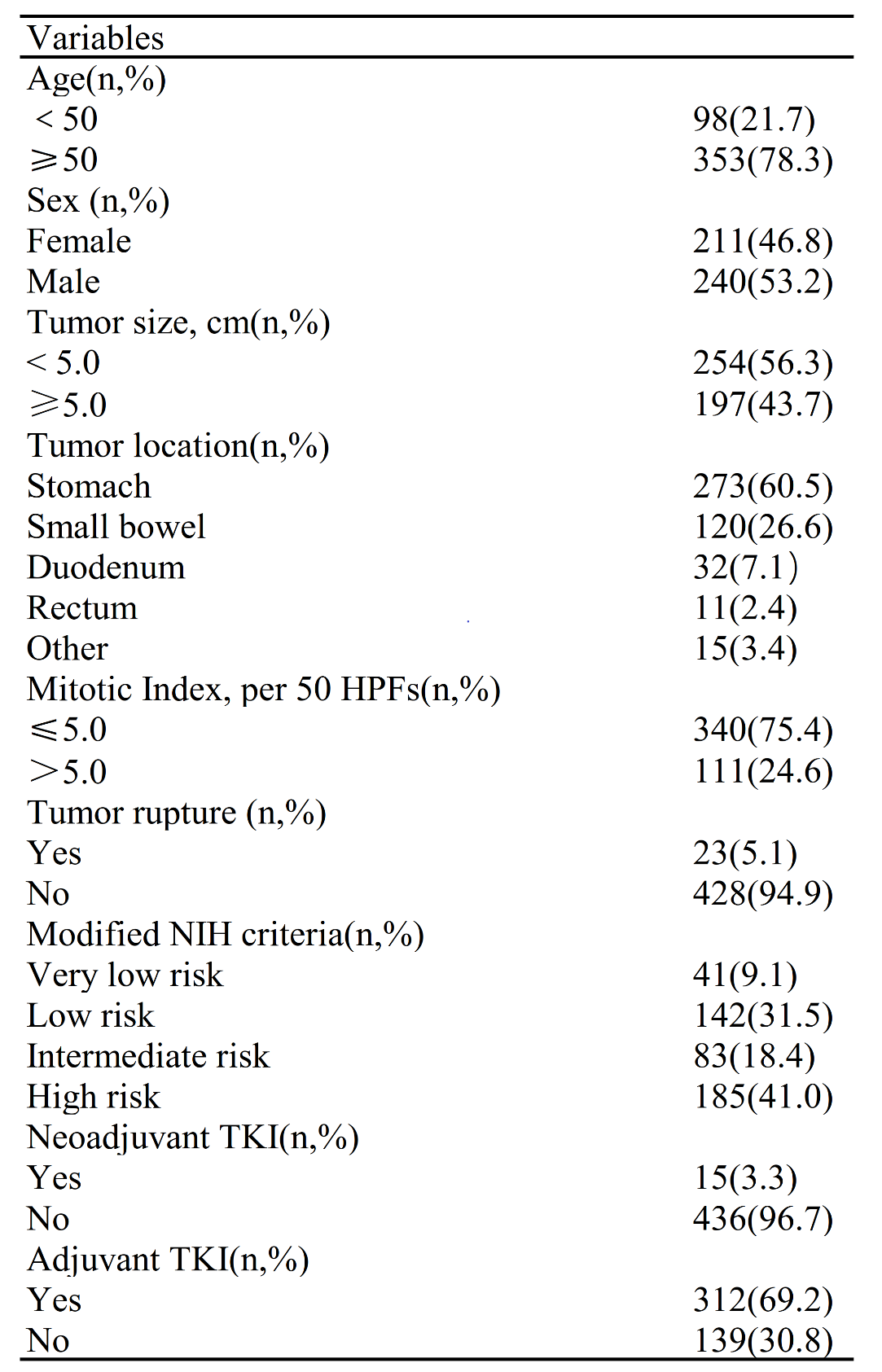


Table S2 . Time-dependent multivariate analysis of the prognostic factors for patients with GISTs


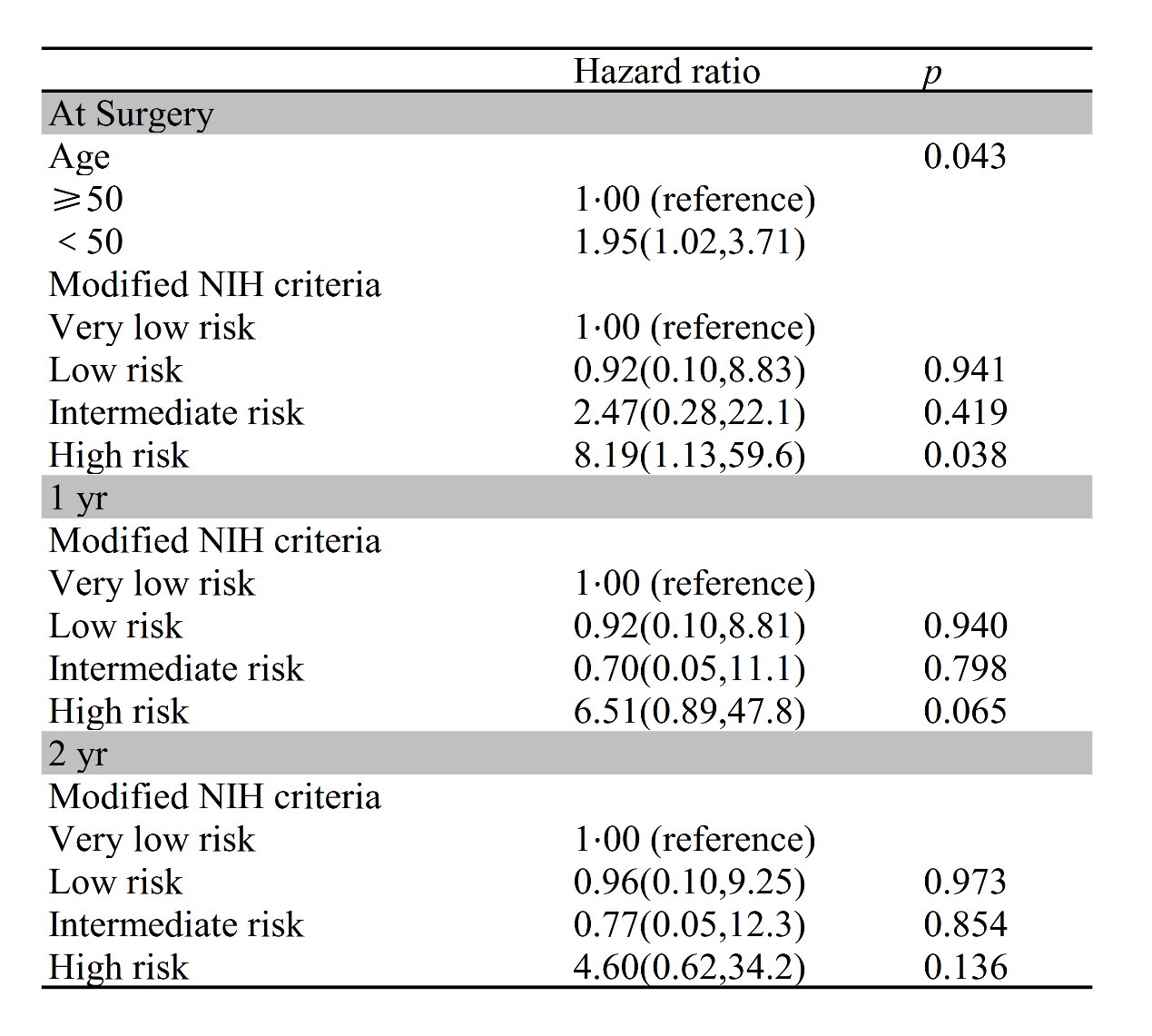


Figure S1. Cohort exclusion criteria.

**
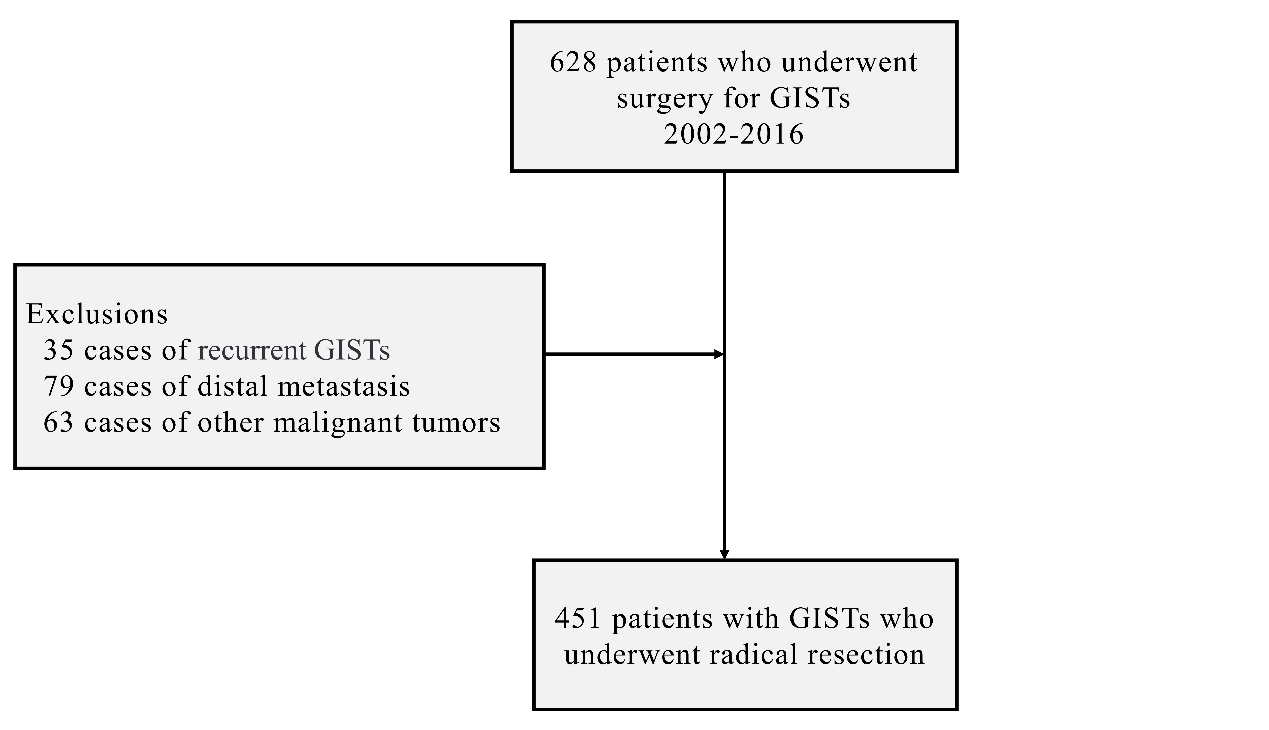
**
